# Injection partnership characteristics and HCV status associations with syringe and equipment sharing among people who inject drugs

**DOI:** 10.1186/s12889-023-16133-5

**Published:** 2023-06-20

**Authors:** Mary Ellen Mackesy-Amiti, Basmattee Boodram, Kimberly Page, Carl Latkin

**Affiliations:** 1 Division of Community Health Sciences, School of Public Health, University of Illinois at Chicago, 1603 W. Taylor St., MC 923, Chicago, IL 60612 USA; 2 Department of Internal Medicine, School of Medicine, University of New Mexico, Albuquerque, NM USA; 3 Department of Health, Behavior, and Society, Bloomberg School of Public Health, Johns Hopkins University, Baltimore, MD USA

**Keywords:** Hepatitis C, Syringe sharing, Network, Partnership, HCV serosorting, People who inject drugs

## Abstract

**Background:**

Sharing of syringes is the leading transmission pathway for hepatitis C (HCV) infections. The extent to which HCV can spread among people who inject drugs (PWID) is largely dependent on syringe-sharing network factors. Our study aims to better understand partnership characteristics and syringe and equipment sharing with those partners, including measures of relationship closeness, sexual activity, and social support, as well as self and partner HCV status to better inform interventions for young urban and suburban PWID.

**Methods:**

Data are from baseline interviews of a longitudinal network-based study of young (aged 18–30) PWID (egos) and their injection network members (alters) in metropolitan Chicago (n = 276). All participants completed a computer-assisted interviewer-administered questionnaire and an egocentric network survey on injection, sexual, and support networks.

**Results:**

Correlates of syringe and ancillary equipment sharing were found to be similar. Sharing was more likely to occur in mixed-gender dyads. Participants were more likely to share syringes and equipment with injection partners who lived in the same household, who they saw every day, who they trusted, who they had an intimate relationship with that included condomless sex, and who provided personal support. PWID who had tested HCV negative within the past year were less likely to share syringes with an HCV positive partner compared to those who did not know their status.

**Conclusion:**

PWID regulate their syringe and other injection equipment sharing to some extent by sharing preferentially with injection partners with whom they have a close personal or intimate relationship, and whose HCV status they are more likely to know. Our findings underscore the need for risk interventions and HCV treatment strategies to consider the social context of syringe and equipment sharing within partnerships.

**Supplementary Information:**

The online version contains supplementary material available at 10.1186/s12889-023-16133-5.

## Introduction

Hepatitis C virus (HCV) infection is a leading cause of chronic liver disease and mortality worldwide [1, 2]. In the United States, 2.4 million people (1% of all adults) are estimated to be living with HCV infection [3]. HCV incidence has been increasing in the U.S., particularly among young (ages 20–39), non-urban people who inject drugs (PWID), consistent with age groups most impacted by the nation’s opioid crisis [4]. Sharing of syringes during injection drug use (IDU) is the leading transmission pathway for HCV infections in many countries including the United States [4]. A meta-analysis of PWID studies in the U.S. from 1997 to 2017 [5] and a recent study among young, predominantly suburban PWID [6] identified network member characteristics that increased risk for syringe sharing among participants, including living in the same household, age, race/ethnicity, gender, having a drug use network member who is also a sexual partner, and HCV status.

The act of sharing injection equipment with another person is impacted by many factors that accumulate to affect network structure and relationship factors that facilitate HIV and HCV transmission [7], including the perceived risk of transmission (e.g., HCV status of oneself and/or injection partner), access to injection equipment, social roles among PWID (e.g., injection, sexual, social support, kin, overlapping [multiplex] roles), relationship closeness, and geographic proximity (e.g., living in same household). Reciprocity is also normative in drug-using relationships [8, 9] and this extends to sharing of syringes and ancillary injection equipment [7]. Psychosocial (e.g., mental health issues, withdrawal, fear of police) and social context (e.g., public injection spaces, social norms) are also linked to sharing injection equipment [10–15]. Social network factors, including network size, composition and structure, have been shown to affect syringe sharing [16], and our recent work shows that it may interact with geography factors such as mobility (e.g., transience) and location of residence (e.g., urban or suburban) [6, 17]. Many studies have also shown serosorting among those aware of their HCV status, where PWID use syringes with seroconcordant individuals (i.e. others with the same infection status) to reduce HIV and HCV transmission risk [18–26]. However, none to our knowledge have included a large proportion of young PWID, the population with the fastest growing incidence in Illinois and highest HCV incidence in the central Midwest.

The influence of these factors on syringe and ancillary injection equipment sharing varies across populations and may change over time. Recent changes in state law^1^ may have resulted in greater access to sterile syringes through pharmacy purchase, however this may also result in fewer PWID accessing syringe service programs and thus less contact with harm reduction services and information. In this paper we examine associations between dyadic partnership characteristics and syringe and ancillary equipment sharing with those partners among young PWID and their IDU networks in the Chicago, Illinois and the surrounding suburban areas to better inform interventions for young urban and suburban PWID. We examine measures of relationship closeness, sexual activity, and social support, as well as perceived self and IDU partner HCV status. In particular, we looked at the interaction of self and partner HCV status to test whether PWID who knew their own status made different decisions about who to share with compared to those who did not know their own status.

## Methods

The data for this study come from baseline interviews conducted from October 2018 to February 2021 for an on-going longitudinal network-based study of young adult (aged 18–30) PWID participants (egos) and their injection network members (alters) [27]. Ego and alter participants completed identical interviews, but only egos were asked to recruit network members.

### Eligibility

To be eligible, ego participants (i.e., initial participants who were asked to recruit their network members) had to be *(i)* 18–30 years old, *(ii)* current injectors (i.e. injected ≥ 1 in past 30 days), *(iii)* willing to recruit their injection network alters who were ≥ 18 years old at baseline, (*iv)* willing to be tested for HIV and HCV, and *(vii)* residing in the Chicago Metropolitan Statistical Area in the past 12 months. The injection network members (i.e. alters) of the egos were eligible if they were *(i)* ≥ 18 years old, (ii) had injected drugs with the ego in the past 6 months. Current injector status was verified by experienced study staff checking for injection stigmata and, if questionable, using a standardized procedure to evaluate participant knowledge of the injection process. Age was verified with a driver’s license or a state ID card. Project staff offered to assist those without identification in obtaining it.

### Ego recruitment

The study was conducted at two field sites of a community outreach organization (Community Outreach Intervention Projects) located in Chicago, Illinois that has been providing services (e.g., harm reduction and HIV and HCV counseling, testing, and case management) and facilitating research with people who use illicit substances for over 30 years. The field sites are located in areas that have rates above the city’s average for HIV, viral hepatitis, and arrests for drug-related offenses and attract both urban and suburban PWID. We recruited most egos from the syringe services program (SSP). In addition, SSP-recruited participants were screened to ascertain if they obtained syringes at the SSP for other people who reside in the suburbs (i.e. secondary exchange). Those who did were offered a coupon to refer to the study an age-eligible peer who did not use the SSP or purchase/use drugs in Chicago. To encourage peer-recruited PWID to participate, we used an outreach van staffed with an interviewer/ phlebotomist to conduct data collection off-site near the recruit’s residence or other mutually agreed upon locations. Alternative outreach methods targeting non-SSP suburban PWID included direct recruitment in drug market areas and at community fairs using an outreach van, posting fliers at community-based organizations serving PWID, and through online ads/social media. Screening and enrollment of non-SSP PWID from drug market areas were done by two field staff with lived experience of injection drug use who have worked in these areas recruiting for similar studies for many years [6, 28].

### Alter recruitment

At their baseline visit, we asked each ego participant to recruit up to five alters (i.e., people they injected drugs with at least once in the prior six months) using recruitment coupons that provided information about the study and were linked to the recruiting ego via alphanumeric code. Coupons could only be redeemed by alters named by an ego participant during their survey. Data collection from recruited alters was required to occur within 6 months of the ego’s baseline visit.

### Procedures

Participants are defined as egos and their recruited alters; both were administered written informed consent and were given an information sheet. All study procedures were approved by the Institutional Review Board of the University of Illinois at Chicago. All methods were performed in accordance with the Declaration of Helsinki and The Common Rule (45 CFR part 46). All participants received compensation of $20/hour for the interview. Most participants completed the survey within a 2-hour session that included a break (average $50). In addition to hourly compensation for interviews, egos were reimbursed $10 for their time for each referred alter successfully screened. All participants received HIV and HCV testing and counseling. All services available at the community center site (e.g., SSP; HCV and HIV testing, counseling, and case management; and linkage to medical care) were made freely available to all PWID screened, regardless of study enrollment.

All participants completed a baseline computer-assisted interviewer-administered questionnaire, including demographics, substance use, HCV antibody and RNA testing, injection-related behaviors, and other measures. Egocentric network data was collected using touchscreen enabled GENSI software [2016] [29] with a graphical interface that allowed participants to sort software-generated nodes that represented the members of their IDU, sex, and support networks into various categories. Core network members were defined as anyone the participant injected with at the same time (not necessarily sharing) at least once in the last 6 months (injection network), had vaginal, anal, or oral sex within at least once in the past 6 months (sexual network), or received social support from at least once in the past 6 months (support network). To reduce participant burden that may result in degraded data [30], particularly for those that the ego only injected with only once for whom they may not know much about, participants were then asked to identify a core network of up to ten people who the participant had injected with, had sex with, or received social support from more than once in the past six months. The upper limit of 10 was selected since reliable measures of network density and composition are possible with as few as 3–5 alters [31]. In this analysis, we report on the core injection networks.

### Measures

#### Sociodemographics

Participants self-reported their gender, age, race, Hispanic/Latinx ethnicity, and employment, and also reported on these for each core network member. Gender was reported as male, female, or transgender. Race and ethnicity were combined to create an indicator variable with categories non-Hispanic white, non-Hispanic Black, Hispanic, and non-Hispanic other race. Employment was defined as receiving money from a regular job (full or part-time) or self-employment. Participants also reported whether or not they had been homeless in the past six months (“Have you been homeless at all in the last six months? (This includes living on the streets or in a shelter for one or more days)”. Participants were asked to report the location of all residences within the last year within the Chicago metropolitan area, which includes the city of Chicago and surrounding suburbs, spanning 16 counties in northeast Illinois, southeast Wisconsin, and northwest Indiana. Our prior work showed significant differences in risk based on region of residence [6, 17, 32]; therefore, we classified participants similarly in these analyses. If all reported residential locations were within Chicago, the individual was classified as urban; if all locations were outside Chicago, they were labeled as suburban. Participants who reported residential locations both within and outside Chicago were labeled as “crossover” transient participants.

#### Closeness

Measures of closeness between the participant and the core injection alters included physical distance, frequency of contact, and trust. Physical distance was assessed by asking “How far do you live from this person” (same household, within my neighborhood, another area of my town/city, outside of my town/city, and out of state). The last two categories were collapsed due to low frequency of “out of state” responses. Frequency of contact was assessed by asking “How often do you talk to or see this person?” with options on a 6-point scale: (1) every day, (2) a few times a week, (3) a few times a month, (4) once a month, (5) a few times a year, (6) less than once a year. We collapsed the last 3 categories into once a month or less. Trust was measured on a 10-point scale, with 1 as “don’t trust at all” and 10 as “trust with my life”.

#### Perceived HCV status

Participants were asked when they were last tested for HCV (antibody and/or RNA), and what was the result of their last test. Participants were categorized as positive at last test, negative within the past 12 months, or unknown if they were never tested, did not know the result of their last test, or received a negative result more than 12 months ago. For each core injection alter, participants were also asked “What is the hepatitis C status of this person?” with options negative, positive, and don’t know.

#### Sexual activity

For core alters, participants were asked how often they engaged in condomless vaginal or anal sex (never, less than half the time, half the time or more, all the time). Responses were recoded as always used a condom or did not always use a condom.

#### Support

For core alters, social support included both *emotional or informational* support (“anyone you could talk to about things that are personal and private or get advice from if a situation came up”) and *shelter* support (“anyone that would let you stay at their place if needed”). Participants were also asked if this was someone who would provide *physical assistance* (“would give you some of their time and energy to help you”), or *material aid* (“would lend or give you $25, or more, or something that was valuable”), if they were someone who they would *trust with money* (e.g. to cash a check or buy groceries), someone they could get together with *socially*, or someone they could ask for *health advice* including for issues such as STIs, birth control, or HIV.

#### Syringe and equipment sharing

For each core injection alter, participants were asked “How often do you share syringes/needles with this person?” and “How often have you shared cookers, cotton, or rinse water with this person?” with responses of daily, weekly, monthly, less than once a month, never, or does not inject drugs. Non-IDU injection partners indicated by “does not inject” were excluded from the analysis. Responses were recoded into binary outcome variables of any syringe sharing and any ancillary equipment sharing.

### Analysis

We compared the sociodemographic characteristics of participants and their injection partners using mixed effects linear and robust (modified) Poisson regression with random subject intercepts to account for dependence between egos and alters. We conducted analyses of syringe and equipment sharing using multilevel mixed effects robust Poisson regression analyses. We tested models with random intercepts for participant and recruitment cluster (i.e. dyad-level observations nested within participant, nested within recruitment clusters). Models were estimated with 12 points of integration. We first examined a set of potential confounders typically associated with syringe and ancillary equipment sharing including participant homelessness, participant and injection partner gender, age, and race-ethnicity, and the interaction of participant gender and injection partner gender. We also included injection partner’s primary method of drug use (IDU or non-IDU).

Next, we tested the effects of the closeness measures, condomless sex, support measures, and participant and injection partner HCV status, and an interaction term to test whether the effect of participant HCV status was modified by partner HCV status, adjusted for participant and partner demographic characteristics. We computed marginal contrasts for multi-category predictor variables, and we computed marginal effects to assess significant interactions. We then estimated a multivariate model to examine the associations of perceived self and partner HCV status, adjusting for demographic and relationship variables. We retained theoretically important covariates selected *a priori* (i.e. participant sociodemographics) regardless of statistical significance. Relationship covariates were selected based on bivariate associations and considering multicollinearity.

## Results

### Characteristics of participants and injection partners

We conducted interviews and collected network data from a total of 323 PWID (who injected drugs in the past six months), excluding duplicate participants. We excluded from the analysis those who reported no core IDU network (n = 10, 3%), leaving 313 individuals who reported on 996 injection partners. Heroin is the most commonly injected drug in the local population, and in this sample 99% injected heroin in the past six months, most (94%) at least weekly. Table 1 shows participant (ego and alter) and injection partner demographic characteristics and injection risk behaviors. Distributions of other injection partner covariates can be found in the supplementary materials (Additional File 1, Table S1). On average, participants reported 3.7 core injection partners. The majority of participants had at least one residence in suburban areas in the past year (30% suburban and 33% crossover) compared to Chicago only residents (35%). Injection partners of suburban and urban residents resided in similar regions; however, only a few (8%) injection partners of crossover transients were also crossover transients, indicating that this group is linking regional networks. Participants were mostly male (73%), as were injection partners (70%). Injection partners tended to be older, and less likely to be employed. Of those who reported sharing a syringe with at least one injection partner in the past 6 months (n = 137, 44%), 84% reported any receptive syringe sharing in the past six months (i.e. 16% may have only engaged in distributive sharing). Conversely, of those who did not report sharing syringes with any of their injection partners (56%), 14% nonetheless reported receptive syringe sharing in the past six months.

**Table 1:** Characteristics of participants (n = 313) and injection partners (n = 996)

	participant		injection partner		contrast^‡^	
	n	%	n	%	z	p-value
**Gender**						
Male	229	73%	693	70%		
Female	83	27%	302	30%	-1.94	0.053
Transgender	1	0.3%	1	0.1%		
**Race/ethnicity**						
NH white	195	62%	680	68%	-1.37	0.172
NH Black	22	7%	115	12%	-1.68	0.093
Hispanic	79	25%	173	17%	2.22	0.026
NH other	17	5%	28	3%		
**Age**						
18–29	198	63%	438	44%		
30–39	88	28%	381	38%		
40 +	27	9%	177	18%		
mean (SD)	30.8 (7.4)		32.5 (8.3)		-3.26	0.001
range	[18–64]		[18–67]			
**Employed**						
No	148	47%	699	70%		
Yes	165	53%	277	28%	7.04	< 0.001
**Homeless past six months**						
No	109	35%	NA		-	
Yes	204	65%				
**Past year residence**						
Chicago only (Urban)	110	35%	395	40%	- ^†^	
Outside Chicago only (Suburban)	95	30%	313	31%		
Crossover transient^a^	104	33%	76	8%		
Missing	4	1%	212	21%		
**Unhoused past year** ^**b**^						
No	168	54%	518	52%	- ^†^	
Yes	141	45%	222	22%		
Missing	4	1%	256	26%		
**Reported HCV status** ^**c**^						
Positive	75	24%	210	21%	1.61	0.108
Negative	128	41%	544	55%	-3.30	0.001
Unknown/not tested past year	110	35%	241	24%	2.52	0.012
**Receptive syringe sharing** ^**d**^						
No	176	56%	NA		-	
Yes	137	44%				
**Ancillary equipment sharing** ^**d**^						
No	72	23%	NA		-	
Yes	240	77%				
**Shared syringe with**:	**any partner**		**this partner**			
No	173	55%	727	73%	NA	
Yes	140	45%	269	27%		
**Shared equipment with**:^**e**^						
No	100	32%	497	50%	NA	
Yes	213	68%	486	49%		

^a^ Past year residence in both urban and suburban areas

^b^ Residence reported as shelter, abandoned building, or on the street/in a car, etc

^c^ Participant baseline self-reported result of last HCV test coded unknown if negative test result was received more than 1 year ago

^d^ any in the past six months

^e^ n = 311 participants, 983 injection partners

^‡^ mixed effects regression contrasting self vs. proxy report

^†^ not computed due to large number of missing

A similar proportion of participants reported a positive HCV status for themselves (25%) and their injection partners (21%); however, while 35% of participants reported unknown/not tested status, only 24% of injection partners were reported as unknown. Among participants who reported a positive HCV test result (n = 75), 45% were tested within the past six months, and 35% more than one year prior. Among those who reported a negative test result (n = 197), 47% were last tested within the past six months, and 35% were tested more than one year ago (these being classified as unknown status.) Participants who self-reported HCV positive were more likely to report an HCV positive injection partner compared to participants who were self-reported HCV negative or unknown (OR = 4.74, 95% CI 2.87–7.82), with 64% reporting at least one HCV positive partner (vs. 27% and 24%), and more than 40% of their injection network identified as HCV positive compared to 14% among HCV negative and unknown.

### Syringe and equipment sharing models

We computed intraclass correlations (ICC) to assess clustering of syringe and equipment sharing within participant (ICC syringe = 0.64, ICC equipment = 0.67) and within recruitment clusters (ICC syringe = 0.23, ICC equipment = 0.12). Likelihood ratio tests indicated that recruitment-level clustering was significant for syringe sharing (LR chi2(1) = 6.35, p = 0.01), but not for equipment sharing (LR chi2(1) = 1.46, p = 0.23).

#### Participant and partner demographics

Table 2 shows the results of mixed effects regressions on syringe and equipment sharing with demographic covariates. There was a strong interaction of participant and injection partner gender in both models (syringe sharing chi2(1) = 14.85, p = 0.0001; equipment sharing chi2(1) = 11.79, p = 0.0006). Male participants were significantly more likely to share syringes with a female partner than with a male partner (marginal predicted prevalence (MPP) 0.36 vs. 0.18; z = 5.44, p < 0.001); and to a lesser degree ancillary equipment (MPP 0.55 vs. 0.42; z = 3.00, p = 0.003). Female participants showed no differentiation by partner gender for syringe sharing (i.e., they shared syringes equally with male and female partners), but were less likely to share injecting equipment with a female than with a male partner (MPP 0.65 vs. 0.50; z = 2.18, p = 0.029).

**Table 2:** Mixed effects^a^ robust Poisson regression on syringe and equipment sharing with injection partners, base demographics

	Shared a syringe^a^				Shared injection equipment^b^			
Predictor variable	aRR	95% Conf. Int.		p	aRR	95% Conf. Int.		p
Age	0.98	0.95	1.01	0.109	0.99	0.97	1.00	0.114
Race-ethnicity:								
non-Hispanic Black	1.00	0.49	2.03	0.999	0.94	0.52	1.71	0.850
Hispanic	0.88	0.63	1.22	0.438	1.03	0.84	1.26	0.781
Other, non-Hispanic	1.50	0.87	2.61	0.146	1.10	0.72	1.67	0.673
*vs. non-Hispanic white*								
Homeless	0.97	0.71	1.33	0.863	1.20	0.99	1.46	0.069
Gender female vs. male/other^c^	2.01	1.39	2.90	0.000	1.48	1.22	1.80	0.000
Partner gender female vs. male/other^c^	2.00	1.55	2.58	0.000	1.29	1.09	1.53	0.003
Gender female x partner gender female	0.41	0.27	0.63	0.000	0.60	0.44	0.81	0.001
Partner primary drug method IDU	2.19	1.17	4.10	0.015	1.93	1.22	3.05	0.005
*vs. non-IDU*								
Partner age	1.00	0.98	1.01	0.631	0.99	0.98	1.00	0.090
Partner race-ethnicity:								
non-Hispanic Black	0.57	0.33	1.00	0.050	0.66	0.47	0.93	0.017
Hispanic	1.01	0.78	1.32	0.913	1.03	0.85	1.25	0.774
Other, non-Hispanic	1.43	0.90	2.26	0.129	0.91	0.61	1.37	0.665
*vs. non-Hispanic white*								
Random intercepts	*Var*	*SE*	*95% Conf. Int.*		*Var*	*SE*	*95% Conf. Int.*	
*recruitment cluster*	0.27	0.11	0.12	0.61	-			
*participant*	0.13	0.12	0.02	0.84	0.02	0.04	0.00	0.81

aRR: adjusted risk ratio

^a^ 3-level model with random intercepts for participant and recruitment cluster; n = 313, 197 clusters, 996 observations

^b^ 2-level model with random intercepts for participant; n = 311, 196 clusters, 983 observations

^c^ 1 transgender person of unknown sex included

#### Injection partnership characteristics

Regression results for partnership characteristics adjusted for demographic variables are shown in Table 3. Unadjusted results are available in supplementary materials (Additional File 1, Table S2). Demographic adjustment had little effect on estimates. Distance was significantly negatively associated with both syringe sharing (Chi2[3] = 23.30, p < 0.0001) and equipment sharing (Chi2[3] = 21.31, p < 0.0001). However, the effect was entirely (syringe sharing) or mostly (other equipment) due to greater sharing of syringes and equipment with injection partners who lived in the “same household” vs. elsewhere (marginal predicted probabilities (MPP) for syringes, 0.41 vs. 0.23; equipment, 0.64 vs. 0.46). Frequency of contact was positively associated with syringe sharing (Chi2(3) = 30.37, p < 0.0001) and equipment sharing (Chi2(3) = 16.52, p = 0.0009), with those who met or talked every day compared to a few times a week significantly more likely to report sharing syringes (MPP = 0.35 vs. 0.25) or equipment (0.60 vs. 0.44).

**Table 3:** Demographic-adjusted effects of injection partnership characteristics and HCV status on syringe and other injection equipment sharing, mixed effects robust Poisson regressions ^a^

	Syringe sharing^b^						Equipment sharing^c^				
Predictor variable	aRR	Robust SE	95% Conf. Int		p		aRR	Robust SE	95% Conf. Int		p
Distance											
live in same household	1.84	0.31	1.33	2.54	< 0.0001		1.21	0.12	0.99	1.47	0.056
within my neighborhood	1.23	0.23	0.86	1.77	0.2590		0.90	0.09	0.73	1.11	0.323
Another area of town	0.82	0.16	0.56	1.20	0.3140		0.76	0.09	0.60	0.95	0.016
*vs. outside of my town/city*											
Frequency of contact											
every day	2.23	0.51	1.43	3.49	0.0000		1.46	0.21	1.10	1.93	0.008
few times a week	1.57	0.36	1.01	2.45	0.0460		1.07	0.16	0.80	1.44	0.633
few times a month	1.14	0.30	0.68	1.91	0.6300		1.07	0.16	0.80	1.42	0.642
*vs. once a month or less*											
Trust rating	1.13	0.02	1.09	1.17	< 0.0001		1.07	0.01	1.05	1.10	< 0.0001
Sex partner	2.42	0.34	1.83	3.19	< 0.0001		1.53	0.13	1.29	1.82	< 0.0001
Condomless sex	2.77	0.38	2.11	3.63	< 0.0001		1.52	0.13	1.28	1.79	< 0.0001
Personal support	2.16	0.26	1.70	2.74	< 0.0001		1.54	0.12	1.33	1.80	< 0.0001
Stay support	1.21	0.19	0.90	1.64	0.2030		1.25	0.10	1.06	1.47	0.009
Material aid	2.02	0.23	1.61	2.53	< 0.0001		1.47	0.11	1.26	1.71	< 0.0001
Physical assistance†	1.95	0.23	1.54	2.46	< 0.0001		1.47	0.12	1.26	1.72	< 0.0001
Financial trust	1.89	0.23	1.49	2.41	< 0.0001		1.43	0.12	1.22	1.68	< 0.0001
Social partner	1.96	0.23	1.56	2.47	< 0.0001		1.47	0.11	1.27	1.72	< 0.0001
Health advice	2.03	0.25	1.60	2.57	< 0.0001		1.56	0.12	1.34	1.81	< 0.0001
**Ego & Alter HCV Status**											
HCV status, last test											
Positive	1.53	0.32	1.01	2.31	0.0440		1.33	0.16	1.05	1.68	0.018
Don’t know or never tested	1.42	0.28	0.97	2.08	0.0740		1.07	0.13	0.85	1.35	0.567
*vs. Negative*											
Alter HCV status											
Positive	1.45	0.17	1.15	1.83	0.0020		1.16	0.11	0.96	1.40	0.130
Don’t Know	0.56	0.13	0.36	0.88	0.0130		0.85	0.11	0.66	1.10	0.222
*vs. Negative*											
**Ego x Alter HCV Status**											
HCV status, last test											
Positive	1.02	0.32	0.56	1.87	0.9430		1.11	0.20	0.78	1.57	0.566
Don’t know or never tested	1.26	0.25	0.85	1.87	0.2520		1.13	0.14	0.90	1.43	0.298
*vs. Negative*											
Alter HCV status											
Positive	0.85	0.24	0.49	1.49	0.5800		0.99	0.18	0.69	1.42	0.960
Don’t Know	0.50	0.22	0.21	1.20	0.1210		0.92	0.18	0.62	1.36	0.678
*vs. Negative*											
HCV status x Alter HCV											
Positive x Positive	2.68	1.05	1.25	5.76	0.0120		1.59	0.40	0.97	2.61	0.065
Positive x Don’t Know	0.99	0.75	0.22	4.42	0.9860		0.89	0.30	0.46	1.74	0.740
Don’t know x Positive	1.47	0.50	0.75	2.88	0.2630		0.78	0.22	0.45	1.36	0.382
Don’t know x Don’t Know	1.28	0.70	0.44	3.76	0.6490		0.89	0.25	0.51	1.56	0.681

aRR: adjusted risk ratio

^a^ adjusted for participant and partner age, race/ethnicity, gender (including interaction), participant homelessness, and partner primary method of drug use

^b^ 3-level model with random intercepts for participant and recruiter-cluster; n = 313, 197 clusters, 995 obs

^c^ 2-level model with random intercepts for participant; n = 311, 982 obs

† dropped homelessness on syringe sharing due to non-convergence

For both syringe and ancillary equipment sharing, the effects of participant and injection partner gender were no longer significant after including being in a sexual relationship. Syringe sharing was more likely in a relationship that included condomless sex, while equipment sharing was simply more likely with a sex partner. Syringe and equipment sharing were more likely with injection partners who provided personal support, but only equipment sharing was more likely with those who would provide a place to stay. All specific aspects of social support (material aid, physical assistance, financial trust, social partner, health advice) were associated with syringe and equipment sharing; however, all were also collinear with personal support.

**Fig. 1:**
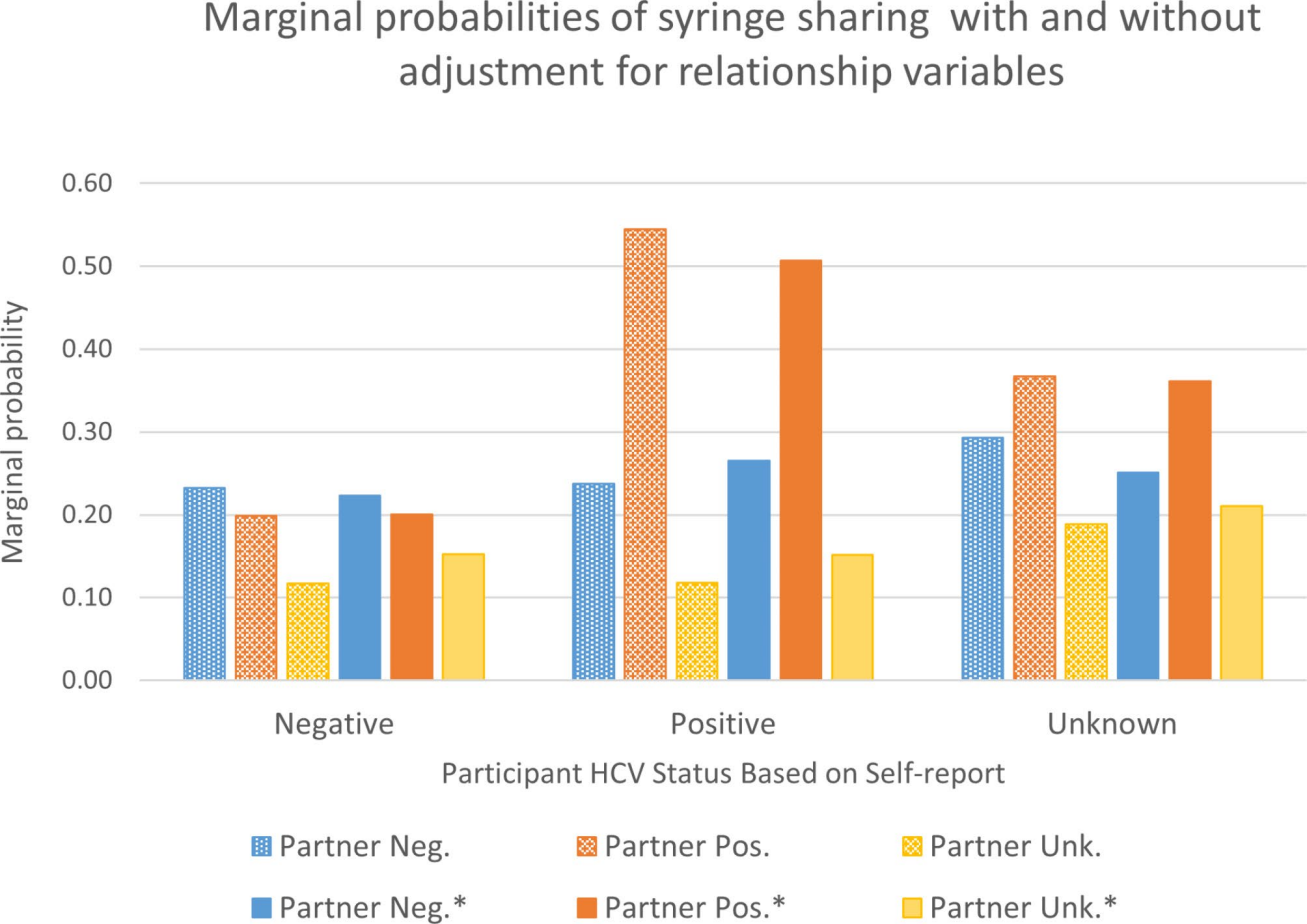
Marginal (adjusted) predictions of syringe sharing with partner by reported participant and injection partner HCV status from multivariable mixed effects robust Poisson regression model with demographic covariates only and (*) with demographic and relationship covariates

*3.2.3. HCV status*. The main effect of partner HCV status (Chi2(2) = 24.42, p < 0.0001) would suggest that PWID are more likely to share syringes with HCV-positive partners than with HCV-negative partners, and less likely to share with HCV status unknown partners. However, there was an interaction between participant and injection partner HCV status (Chi2(4) = 12.21, p = 0.016); planned contrasts indicated that the effect of injection partner HCV status varied according to participant HCV status (Chi2(6) = 37.29 p < 0.0001) and the effect of participant HCV status varied according to injection partner HCV status (Chi2(6) = 17.44, p = 0.0078). Marginal (adjusted) predictions are shown in Fig. 1. Participants who had tested positive were more likely to share syringes with an injection partner who was also positive (MPP = 0.54), both compared to participants who had tested negative (MPP = 0.20, Chi2[1] = 14.16, p = 0.0002), and compared to sharing with an injection partner who was perceived to be HCV negative (MPP = 0.24, Chi2[1] = 15.12, p = 0.0001.) All participants were least likely to share syringes with a partner of unknown HCV status.

For equipment sharing, there was a similar interaction between participant and injection partner HCV status (Chi2[4] = 13.01, p = 0.011). Planned contrasts indicated that the effect of injection partner HCV status varied according to participant HCV status (Chi2(6) = 18.62 p = 0.0049) and the effect of participant HCV status varied according to injection partner HCV status (Chi2(6) = 24.77, p = 0.0004). Participants who had tested positive were more likely to share equipment with an injection partner who was also positive (MPP = 0.79), both compared to participants who had tested negative (MPP = 0.45, Chi2[1] = 12.56, p = 0.0004), and compared to sharing with an injection partner who was perceived to be HCV negative (MPP = 0.50, Chi2[1] = 9.48, p = 0.0021.)

**Fig. 2:**
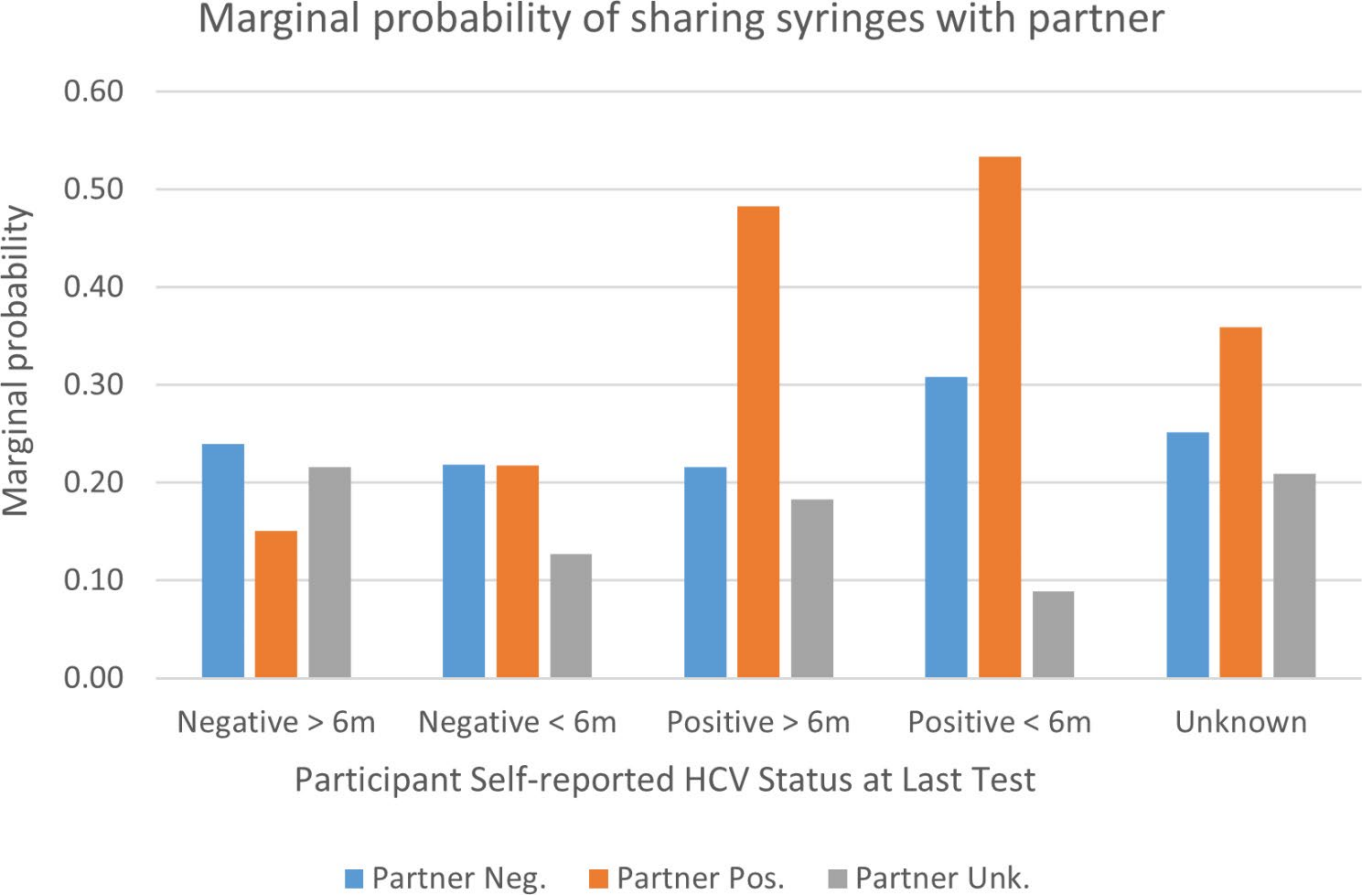
Marginal (adjusted) probabilities of syringe sharing with partner by reported participant and injection partner HCV status and timing of HCV test result (within past 6 months or not) based on multivariable mixed effects robust Poisson regression model with demographic and relationship covariates

In a supplemental analysis, we looked at whether the timing of HCV diagnosis or last negative test (within past six months vs. more than six months ago) affected the association between self and partner HCV status and sharing syringes and equipment. Regardless of whether they had received the test result within the past six months or prior, participants reporting a positive HCV status were about twice as likely to have shared syringes with an alter they knew to be HCV positive vs. negative, while participants who had tested negative showed little variation in partner status regardless of timing (Fig. 2).

#### Final models

The final models including demographic and selected relationship characteristics are shown in Table 4. Marginal (adjusted) predictions for syringe sharing are shown in Fig. 1. In both models, the HCV status interaction was weaker (syringes: Chi2[4] = 9.51, p = 0.05; equipment: Chi2[4] = 9.52, p = 0.049) after adjusting for partner characteristics. Marginal contrasts indicated that the effect of injection partner HCV status on syringe sharing varied according to participant HCV status (Chi2(6) = 24.26, p = 0.001). Participants who had tested positive were more likely to share syringes with an injection partner who was also positive (MPP = 0.51), compared to participants who had tested negative (MPP = 0.20, Chi2[1] = 13.50, p = 0.0002), and compared to sharing with an injection partner who was perceived to be HCV negative (MPP = 0.27, Chi2[1] = 10.90, p = 0.001.) Sharing with HCV negative and unknown status partners did not vary significantly by participant HCV status.

**Table 4:** Multivariable mixed effects^a^ robust Poisson regression on syringe and other injection equipment sharing with injection partners

	Syringe sharing^a^					Equipment sharing^b^				
Predictor variable	aRR	Robust SE	95% Conf. Int.		p	aRR	Robust SE	95% Conf. Int.		p
Age	0.97	0.02	0.94	1.01	0.145	0.98	0.01	0.97	1.00	0.074
Gender female vs. male/other^c^	1.29	0.22	0.92	1.80	0.139	1.19	0.12	0.97	1.45	0.096
Partner gender female vs. male/other^c^	1.25	0.12	1.03	1.51	0.022	1.00	0.07	0.87	1.15	0.993
Race-ethnicity:										
non-Hispanic Black	1.32	0.51	0.62	2.80	0.475	1.02	0.29	0.58	1.79	0.949
Hispanic	0.91	0.15	0.66	1.26	0.580	1.06	0.11	0.86	1.31	0.570
Other, non-Hispanic	1.14	0.34	0.63	2.06	0.666	0.95	0.19	0.63	1.41	0.782
*vs. non-Hispanic white*										
Homeless	0.98	0.15	0.72	1.33	0.883	1.22	0.12	1.01	1.48	0.042
Partner age	1.00	0.01	0.99	1.02	0.651	0.99	0.01	0.98	1.00	0.153
Partner race-ethnicity										
non-Hispanic Black	0.63	0.15	0.39	1.01	0.053	0.69	0.11	0.50	0.94	0.020
Hispanic	1.05	0.14	0.81	1.35	0.720	1.03	0.10	0.85	1.25	0.738
Other, non-Hispanic	1.42	0.32	0.91	2.20	0.119	0.86	0.15	0.61	1.21	0.382
*vs. non-Hispanic white*										
Partner method IDU vs. non-IDU	2.02	0.61	1.11	3.65	0.020	1.84	0.40	1.19	2.83	0.006
Live in same household	1.07	0.13	0.85	1.35	0.548	1.10	0.10	0.91	1.32	0.322
Trust rating	1.05	0.02	1.01	1.10	0.009	1.04	0.02	1.01	1.07	0.008
Sex partner	-					1.24	0.11	1.04	1.48	0.017
Condomless sex	1.87	0.23	1.47	2.38	0.000					
Personal support	1.38	0.19	1.06	1.79	0.017	1.26	0.11	1.05	1.50	0.012
HCV status, last test										
Positive	1.19	0.35	0.67	2.12	0.560	1.16	0.21	0.81	1.65	0.426
Unknown or not tested	1.12	0.24	0.74	1.70	0.581	1.06	0.13	0.83	1.35	0.653
*vs. Negative in past 12 months*										
Alter HCV status										
Positive	0.90	0.24	0.53	1.52	0.691	1.03	0.18	0.73	1.46	0.877
Don’t Know	0.68	0.30	0.29	1.62	0.387	1.08	0.22	0.73	1.60	0.689
*vs. Negative*										
HCV status x Alter HCV										
Positive x Positive	2.13	0.77	1.05	4.32	0.037	1.45	0.36	0.89	2.36	0.139
Positive x Don’t Know	0.84	0.60	0.21	3.38	0.802	0.85	0.28	0.45	1.62	0.623
Don’t know x Positive	1.60	0.54	0.83	3.08	0.160	0.81	0.22	0.48	1.39	0.447
Don’t know x Don’t Know	1.23	0.65	0.44	3.45	0.697	0.89	0.25	0.52	1.53	0.671
Random intercepts	*Var*	*SE*	*95% Conf. Int.*			*Var*	*SE*	*95% Conf. Int.*		
*recruitment cluster*	0.15	0.10	0.04	0.53		-				
*participant*	0.13	0.13	0.02	0.99		8.39E-32	2.88E-31	1.01E-34	6.97E-29	

^a^ 3-level model with random intercepts for participant and recruitment cluster; n = 313, 197 clusters, 995 obs

^b^ 2-level model with random intercepts for participant; n = 311, 982 obs

^c^ 1 transgender person of unknown sex included

For equipment sharing, adjusting for relationship characteristics, marginal contrasts indicated that the effect of participant HCV status varied over injection partner status (Chi2[6] = 24.24, p = 0.0005). Participants who had tested positive were more likely to share equipment with an injection partner who was also positive (MPP = 0.76), compared to participants who had tested negative (MPP = 0.46, Chi2[1] = 11.59, p = 0.0007), and compared to sharing with an injection partner who was perceived to be HCV negative (MPP = 0.51, Chi2[1] = 7.01, p = 0.008.)

## Discussion

Our study of young urban and suburban PWID from a large metropolitan area examined interpersonal and network factors within injection drug use dyads on syringe and ancillary equipment sharing and HCV status. As in previous studies [5, 33–36] younger PWID were more likely to engage in risky injection practices. Our study supports others showing that syringe sharing is common among mixed-gender dyads within the context of a close sexual relationship (e.g., condomless sex) [37–41], while equipment sharing was simply more likely in a sexual relationship. Similar to other studies on PWID, closeness and trust were important interpersonal factors [40, 42, 43]; PWID in our study were more likely to share syringes with those who they trusted and who provided personal support.

As in our prior study of young PWID [6], participants were more likely to share syringes and equipment with injection partners who lived in the same household and who they saw every day. However, in the fully adjusted model, trust and personal support remained strong predictors of both syringe and equipment sharing, while living in the same household did not. This finding highlights the importance of relationship quality over proximity. Our study reports on a novel and under-reported population in the PWID literature—young PWID residing in suburban areas (63% of the sample reported at living in a suburban area in the prior year). Notably, closeness within injection dyads of young suburban PWID with close sexual relationships may not necessarily translate to living within close proximity, much less within the same household. While PWID who resided exclusively in urban areas (Chicago, Illinois) in the prior year also reported meeting sexual partners predominantly in Chicago, suburban and crossover PWID in this study (data not shown) and our prior study of young PWID [17] reported meeting sexual partners in both urban and suburban areas, i.e., across large distances.

Our study provides support for some degree of serosorting by HCV status as found in other studies of PWID of all ages [18–26], and exhibits the tension between protection and risk within close primary injection partnerships as reported in a recent qualitative study [41]. Participants who tested HCV positive were more likely to share syringes and equipment with HCV positive injection partners compared to those who reported negative HCV status, and compared to sharing with an injection partner who was perceived to be HCV negative. However, adjusting for relationship variables reduced the influence of injection partner HCV status on sharing, possibly indicating relationship variables such as closeness as the more important factor affecting risk management.

Our findings related to the timing of HCV positive tests suggest that the elevated level of syringe and equipment sharing between HCV positive participants and their partners also likely reflects to some degree risk behavior leading to infection. Conceivably, injecting within close partnerships could potentially deter HCV testing if PWID do not see added value as they are already sharing preferentially with injection partners with whom they have a close personal or intimate relationship. Indeed, few PWID in our study seek HCV testing as often as needed (e.g., regularly if engaged in risk activities and antibody negative from an initial test). Overall, only 40% had been tested in the past six months. Our study supports the need for targeted HCV testing and leveraging dyad relationships to reduce the stigma of HCV so that people could disclose HCV to partners to increase protective serosorting [44].

Our study is one of few that have examined the role of injection partnerships and HCV risk among young PWID in the United States [24] and the only one to our knowledge that includes a large proportion of suburban PWID. Overall, we found that PWID regulate their syringe and other injection equipment sharing to some extent by sharing preferentially with injection partners with whom they have a close personal or intimate relationship, and whose HCV status they may be more likely to know. While this likely reflects a correlation between safer injection behavior and HCV testing, it suggests the possibility that HCV testing could incentivize safer behavior given the suboptimal testing of this young population.

Our study has several limitations. First, cross-sectional data were used, so causal inferences are limited. Second, generalizability to all PWID is limited since most of the sample was 18–39 years old, and alter recruitment was restricted to people with whom egos interacted more frequently, which is associated with risk. Finally, self-reported HCV and participant knowledge of partner HCV status is limiting; however, recruitment occurred at community sites located in drug market areas and using a mobile van, all provided free, regular HCV testing so this is likely a relatively highly tested population.

**Conclusion**.

Our study provides insights into the interdependence of interpersonal factors and risk behavior within injection dyads. Our findings underscore the need for intervention strategies to consider the social context of syringe and equipment sharing within partnerships of young PWID. These may include (i) leveraging injection partnerships to increase communication and disclosure of HCV status and sharing practices [44]; (ii) regular HCV rapid testing within partnerships; (iii) counseling on strategies to minimize transmission (e.g., serosorting); and (iv) provision of services by SSPs based partly on injection network size. Given the alarming increases in HCV among young PWID in the United States, further qualitative research is needed to explore the feasibility of dyad-based interventions for young PWID.

## Electronic supplementary material

Below is the link to the electronic supplementary material.

Supplementary Material 1

## Data Availability

The datasets analyzed during the current study are available from the corresponding author on reasonable request.

## Footnotes

1 in 2018 Illinois law was amended to allow the sale of up 100 syringes without a prescription (previously limited to 20).
