# Supplementary material for: Injection partnership characteristics and HCV status associations with syringe and equipment sharing among people who inject drugs
Source: BMC Public Health. 2023 Jun 20;23:1191. doi: 10.1186/s12889-023-16133-5 (PMC10283252; doi:10.1186/s12889-023-16133-5)
Supplement: Supplementary file 1 — Supplementary Material 1 [file 12889_2023_16133_MOESM1_ESM.docx]

**Injection partnership characteristics and HCV status associations with syringe and equipment sharing among people who inject drugs**

M.E. Mackesy-Amiti, B. Boodram, K. Page, C. Latkin

**Table S1. Characteristics of injection partners (N=996) reported by participants (N=313)**

|  | Injection partners | | Participants^a^ | |
| --- | --- | --- | --- | --- |
| Variable | *N* | *%* | *N* | *%* |
| Distance |  |  |  |  |
| Same household | 166 | 17% | 133 | 42% |
| Within my neighbourhood | 323 | 32% | 151 | 48% |
| Another area of town | 281 | 28% | 139 | 44% |
| Outside of my town/city | 221 | 22% | 123 | 39% |
| Missing | 5 | 0.5% | 1 | 0.3% |
| Meet frequency |  |  |  |  |
| every day | 375 | 38% | 230 | 73% |
| few times a week | 327 | 33% | 179 | 57% |
| few times a month | 169 | 17% | 111 | 35% |
| once a month or less | 122 | 12% | 71 | 23% |
| Missing | 3 | 0.3% | 0 | 0% |
| Condomless sex |  |  |  |  |
| no | 841 | 84% | 279 | 89% |
| yes | 155 | 16% | 132 | 42% |
| Personal support |  |  |  |  |
| no | 757 | 76% | 269 | 86% |
| yes | 239 | 24% | 153 | 49% |
| Stay support |  |  |  |  |
| no | 853 | 86% | 293 | 94% |
| yes | 143 | 14% | 103 | 33% |
| Material aid |  |  |  |  |
| no | 833 | 84% | 281 | 90% |
| yes | 163 | 16% | 127 | 41% |
| Physical assistance |  |  |  |  |
| no | 825 | 83% | 281 | 90% |
| yes | 170 | 17% | 129 | 41% |
| Missing | 1 | 0.1% | 1 | 0.3% |
| Financial trust |  |  |  |  |
| no | 839 | 84% | 280 | 89% |
| yes | 156 | 16% | 122 | 39% |
| Missing | 1 | 0.1% | 0 | 0% |
| Social partner |  |  |  |  |
| no | 812 | 82% | 278 | 89% |
| yes | 183 | 18% | 138 | 44% |
| Missing | 1 | 0.1% | 1 | 0.3% |
| Health advice (N=993 / 312) |  |  |  |  |
| no | 841 | 85% | 282 | 90% |
| yes | 152 | 15% | 121 | 39% |
| Missing | 3 | 0.3% | 1 | 0.3% |
|  | *mean* | *SD* | *SD between* | *SD within* |
| Trust rating | 5.49 | 3.15 | 2.54 | 2.24 |
| ^a^ Number and percent of participants who reported at least one injection partner with this characteristic | | | | |

**Table S2. Unadjusted bivariate associations of injection partnership characteristics and HCV status with syringe and ancillary injection equipment sharing, mixed effects robust Poisson regression^a^**

|  | **Syringe sharing^a^** | | | | |  | **Equipment sharing^b^** | | | | |
| --- | --- | --- | --- | --- | --- | --- | --- | --- | --- | --- | --- |
| **Predictor variable** | **RR** | **Robust SE** | **95% Conf. Int** | | **p** |  | **RR** | **Robust SE** | **95% Conf. Int** | | **p** |
| Distance |  |  |  |  |  |  |  |  |  |  |  |
| live in same household | 2.03 | 0.35 | 1.45 | 2.84 | < .0001 |  | 1.29 | 0.13 | 1.06 | 1.57 | 0.012 |
| within my neighbourhood | 1.07 | 0.20 | 0.74 | 1.56 | 0.7130 |  | 0.83 | 0.09 | 0.67 | 1.03 | 0.088 |
| Another area of town | 0.74 | 0.15 | 0.50 | 1.10 | 0.1430 |  | 0.73 | 0.09 | 0.58 | 0.93 | 0.009 |
| *vs. outside of my town/city* | |  |  |  |  |  |  |  |  |  |  |
| Frequency of contact |  |  |  |  |  |  |  |  |  |  |  |
| every day | 2.44 | 0.57 | 1.54 | 3.86 | < .0001 |  | 1.52 | 0.22 | 1.15 | 2.02 | 0.003 |
| few times a week | 1.52 | 0.36 | 0.95 | 2.42 | 0.0810 |  | 1.10 | 0.17 | 0.81 | 1.49 | 0.552 |
| few times a month | 1.14 | 0.30 | 0.68 | 1.92 | 0.6110 |  | 1.07 | 0.16 | 0.80 | 1.43 | 0.658 |
| *vs. once a month or less* |  |  |  |  |  |  |  |  |  |  |  |
| Trust rating | 1.15 | 0.02 | 1.11 | 1.20 | < .0001 |  | 1.08 | 0.02 | 1.05 | 1.11 | < .0001 |
| Sex partner | 2.73 | 0.33 | 2.15 | 3.46 | < .0001 |  | 1.64 | 0.11 | 1.43 | 1.88 | < .0001 |
| Condomless sex | 3.12 | 0.37 | 2.47 | 3.95 | < .0001 |  | 1.72 | 0.12 | 1.50 | 1.98 | < .0001 |
| Personal support | 2.48 | 0.32 | 1.93 | 3.20 | < .0001 |  | 1.65 | 0.13 | 1.42 | 1.91 | < .0001 |
| Stay support | 1.26 | 0.19 | 0.94 | 1.70 | 0.1190 |  | 1.31 | 0.11 | 1.11 | 1.55 | 0.001 |
| material aid | 2.43 | 0.30 | 1.91 | 3.08 | < .0001 |  | 1.59 | 0.12 | 1.38 | 1.84 | < .0001 |
| physical assistance | 2.33 | 0.29 | 1.81 | 2.98 | < .0001 |  | 1.59 | 0.12 | 1.37 | 1.85 | < .0001 |
| financial trust | 2.25 | 0.29 | 1.75 | 2.91 | < .0001 |  | 1.55 | 0.12 | 1.33 | 1.80 | < .0001 |
| social partner | 2.31 | 0.29 | 1.81 | 2.95 | < .0001 |  | 1.59 | 0.12 | 1.36 | 1.84 | < .0001 |
| health advice | 2.40 | 0.32 | 1.86 | 3.11 | < .0001 |  | 1.67 | 0.12 | 1.45 | 1.92 | < .0001 |
| **Ego & Alter HCV Status** |  |  |  |  |  |  |  |  |  |  |  |
| HCV status, last test |  |  |  |  |  |  |  |  |  |  |  |
| Positive | 1.46 | 0.32 | 0.95 | 2.25 | 0.0820 |  | 1.26 | 0.16 | 0.98 | 1.62 | 0.069 |
| Don't know or never tested | 1.53 | 0.32 | 1.02 | 2.30 | 0.0410 |  | 1.13 | 0.14 | 0.88 | 1.45 | 0.343 |
| *vs Negative* |  |  |  |  |  |  |  |  |  |  |  |
| Alter HCV status |  |  |  |  |  |  |  |  |  |  |  |
| Positive | 1.44 | 0.18 | 1.14 | 1.83 | 0.0020 |  | 1.21 | 0.12 | 0.99 | 1.47 | 0.057 |
| Don't Know | 0.50 | 0.12 | 0.31 | 0.79 | 0.0030 |  | 0.80 | 0.11 | 0.62 | 1.03 | 0.089 |
| *vs. Negative* |  |  |  |  |  |  |  |  |  |  |  |
| **Ego x Alter HCV Status** |  |  |  |  |  |  |  |  |  |  |  |
| HCV status, last test |  |  |  |  |  |  |  |  |  |  |  |
| Positive | 1.00 | 0.31 | 0.54 | 1.84 | 0.9880 |  | 1.03 | 0.20 | 0.71 | 1.50 | 0.861 |
| Don't know or never tested | 1.44 | 0.32 | 0.93 | 2.23 | 0.1010 |  | 1.18 | 0.16 | 0.90 | 1.54 | 0.222 |
| *vs Negative* |  |  |  |  |  |  |  |  |  |  |  |
| Alter HCV status |  |  |  |  |  |  |  |  |  |  |  |
| Positive | 0.88 | 0.29 | 0.46 | 1.69 | 0.7050 |  | 0.99 | 0.19 | 0.67 | 1.44 | 0.942 |
| Don't Know | 0.49 | 0.22 | 0.21 | 1.19 | 0.1150 |  | 0.86 | 0.18 | 0.57 | 1.29 | 0.459 |
| *vs. Negative* |  |  |  |  |  |  |  |  |  |  |  |
| HCV status x Alter HCV |  |  |  |  |  |  |  |  |  |  |  |
| Positive x Positive | 2.64 | 1.14 | 1.13 | 6.17 | 0.0250 |  | 1.72 | 0.45 | 1.03 | 2.87 | 0.037 |
| Positive x Don't Know | 1.01 | 0.79 | 0.22 | 4.65 | 0.9860 |  | 0.93 | 0.33 | 0.47 | 1.86 | 0.844 |
| Don't know x Positive | 1.33 | 0.53 | 0.61 | 2.89 | 0.4660 |  | 0.84 | 0.26 | 0.47 | 1.53 | 0.577 |
| Don't know x Don't Know | 1.03 | 0.57 | 0.35 | 3.02 | 0.9610 |  | 0.89 | 0.26 | 0.50 | 1.59 | 0.703 |
| ^a^ 3-level model with random intercepts for participant and ego-cluster; n = 313, 197 clusters, 995 obs | | | | | | | | | | | |
| ^b^ 2-level model with random intercepts for participant; n = 311, 982 obs | | | | | | | | | | | |
